# Optogenetics Neuromodulation of the Nose

**DOI:** 10.1155/2024/2627406

**Published:** 2024-08-13

**Authors:** Feng Xiang, Shipeng Zhang, Mi Tang, Peijia Li, Hui Zhang, Jiahui Xiong, Qinxiu Zhang, Xinrong Li

**Affiliations:** ^1^ TCM Department Chongqing University Cancer Hospital Chongqing Cancer Hospital, Chongqing, China; ^2^ E.N.T. Department Hospital of Chengdu University of Traditional Chinese Medicine, Chengdu, China; ^3^ E.N.T. Department Chengdu University of Traditional Chinese Medicine, Chengdu, China

**Keywords:** nose, olfactory neural circuit, optogenetics, trigeminal nerve

## Abstract

Recently developed optogenetic technology, which allows high-fidelity control of neuronal activity, has been applied to investigate the neural circuits underlying sensory processing and behavior. The nasal cavity is innervated by the olfactory nerve and trigeminal nerve, which are closely related to common symptoms of rhinitis, such as impairment of smell, itching, and sneezing. The olfactory system has an amazing ability to distinguish thousands of odorant molecules at trace levels. However, there are many issues in olfactory sensing mechanisms that need to be addressed. Optogenetics offers a novel technical approach to solve this dilemma. Therefore, we review the recent advances in olfactory optogenetics to clarify the mechanisms of chemical sensing, which may help identify the mechanism of dysfunction and suggest possible treatments for impaired smell. Additionally, in rhinitis patients, alterations in the other nerve (trigeminal nerve) that innervates the nasal cavity can lead to hyperresponsiveness to various nociceptive stimuli and central sensitization, causing frequent and persistent itching and sneezing. In the last several years, the application of optogenetics in regulating nociceptive receptors, which are distributed in sensory nerve endings, and amino acid receptors, which are distributed in vital brain regions, to alleviate overreaction to nociceptive stimuli, has gained significant attention. Therefore, we focus on the progress in optogenetics and its application in neuromodulation of nociceptive stimuli and discuss the potential clinical translation for treating rhinitis in the future.

## 1. Introduction

The nasal cavity is innervated by two main structures: the olfactory nerve, which provides the sense of smell, and the trigeminal nerve, which provides the sense of irritation). Nasal symptoms include impaired smell, itching, and sneezing, among others. Most olfactory deficits are caused by neuronal factors. Impaired smell can occur following injury anywhere along the olfactory tract, from the nasal cavity to the brain. If recovery is possible, it usually occurs within 6 months to a year after infection [1]. However, the probability of recovery beyond 2 years postinjury becomes very low. Despite its seemingly harmless presentation, olfactory deficits can have devastating long-term effects that persist long after the initial life-threatening injuries have been resolved or stabilized [2]. Currently, there are no commercially available specific treatments for sensorineural smell loss [3]. In addition, itching and sneezing are two of the main bothersome symptoms in patients with rhinitis, such as allergic rhinitis (AR) or idiopathic rhinitis. The activation of the central and peripheral nervous systems plays a significant role in the pathophysiology of these symptoms. Nasal sensory fibers can be stimulated by products of allergic reactions as well as external physical and chemical irritants, thereby transmitting signals that generate sensations such as itching and motor reflexes like sneezing [4, 5]. These symptoms often recur and significantly impact the quality of life for patients. One study reported that treating rhinitis patients (especially those with non-AR) with capsaicin improved overall nose function compared to a placebo [6]. This effect appeared to be better than another common type of nasal medication, budesonide (a steroid) [6]. It is hypothesized that repeated high doses of capsaicin lead to the degeneration of nerve fibers involved in neurogenic reflex mechanisms in the nasal mucosa, both locally and centrally [6]. Therefore, a comprehensive understanding of the anatomy and pathophysiology is crucial for identifying the mechanisms of dysfunction in the nose and suggesting possible treatments for rhinitis symptoms such as impaired smell, itching, and sneezing.

Optogenetics provides a powerful tool for high-precision neuromodulation, allowing for the control of specific cells modified with photosensitive proteins, opsins, through light pulses of specific wavelengths [7]. The two most commonly used opsins are inhibitory halorhodopsin and excitatory channelrhodopsin, which are also ion channel protein [8]. For instance,*Chlamydomonas reinhardtii*'s channelrhodopsin-2 (ChR2) is a microbial opsin that is activated by blue light (~480 nm) and rapidly opens the ion channel. This allows cations to enter the neural cell through its membrane, leading to depolarization and subsequent neuronal firing [9]. Another important rhodopsin that restricts neuronal activity is halorhodopsin (NpHR), derived from a halophilic bacterium [9]. NpHR functions as a chloride pump and responds to yellow light (~590 nm) by allowing chloride ions to pass through cell membranes [10]. This mechanism causes neurons to hyperpolarize. Exogenous opsin is transfected and expressed in specific neurons using adenovirus or lentivirus as vectors, enabling the regulation of only the desired neurons. The introduction of opsin allows these originally light-insensitive neurons to respond to light through depolarization or hyperpolarization. By manipulating the activity of neurons using optogenetics, researchers can gain a deeper understanding of the mechanisms underlying brain plasticity. This knowledge is crucial for elucidating the potential structure and function of neural circuits involved in nasal control. In recent years, there has been widespread interest in the application of optogenetics in studying olfactory function mechanisms [11]. This article provides a review of optogenetics and its application in regulating nasal nerve activity, along with a discussion on its potential clinical applications in the future.

## 2. Application in Olfactory Neural Circuits

**Figure 1:**
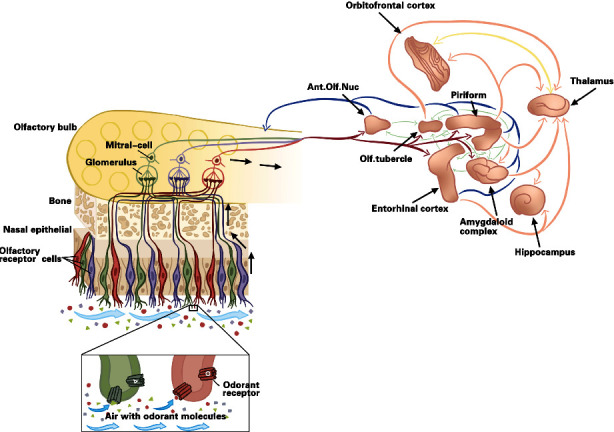
The olfactory system of humans. Connections are demonstrated from the olfactory epithelium to the olfactory bulb, from the olfactory bulb to central brain structures, and from bulbar recipients to higher cortical structures. The regions of central brain structures: projections from the olfactory bulb (red), reciprocal connections to the olfactory bulb (blue), projections from bulbar recipients to higher cortical structures (orange), thalamo-cortical connections (yellow), and connections within bulbar recipients (green). Credit: Karolinska Institutet and Nobel Foundation, Stockholm, Sweden.

Olfaction, or the perception of odors, plays a vital role in human social interaction and everyday life [12, 13]. The olfactory signal transduction pathway primarily encompasses the olfactory receptor neuron (ORN), the olfactory bulb (OB), and the olfactory cortex (Figure 1) [14]. Optogenetics has helped shed light on the intricacies of olfaction. Researchers have cleverly employed light to activate components of the olfactory pathway, such as ORNs or neurons located further downstream, while leaving other brain circuits untouched. This approach aids in understanding how the brain processes smells. The subsequent sections will delve into the application of optogenetics in deciphering how the brain makes sense of smells and discuss its potential clinical applications in the future.

### 2.1. Application in ORN

The use of optogenetics in the first stage of olfactory neural transmission pathways, ORNs, helps to understand the mechanisms by which olfactory information is transformed into navigation behavior. ORNs play an important role in olfactory signal transduction. The odorant receptors are localized on ORNs, which occupy a small area in the upper part of the nasal epithelium. Each ORN expresses only one type of odorant receptor that binds to a specific class of odorant molecule. Each low concentration of an odor only causes a physiological response in its homologous ORN, while high concentrations of each odor can cause physiological responses in multiple ORNs and are usually used for routine behavioral analysis [15–17]. The behavioral patterns induced by low- and high-concentration odors differ, making the interpretation of behavioral research more complex due to this odor variability [18, 19]. The advantages of optogenetics depend on spatial and temporal precision, which allows the activation of individual olfactory receptor cells. In addition, changing the frequency of light stimulation enables us to change the timing pattern of ORN activation, which is difficult to achieve using odor stimulation.

Hernande-Nunez et al. found that light-induced activation of two independent ORNs, expressing Or42a and Or45a, respectively, could trigger pulse sequences in single sensory neurons and evoke navigational responses (run and turn movements) in larvae [20]. In addition, studies have shown that photostimulation of olfactory sensory neurons and OBs expressing the olfactory marker protein (OMP)/ChR2 mouse model can induce electro-olfactograms. The electro-olfactograms induced by light stimulation are weaker and shorter than that induced by odor stimulation of the same duration. The reason is that the responses induced by odor and light are derived from distinct regions of the OSN. Odor transduction occurs in the chemosensory cilia, while light responses originate outside the cilia and do not activate ciliary Ca2+-gated Cl-channels. Furthermore, the electro-olfactograms induced by light stimulation of different durations, intensities, and frequencies differ. The response of the epithelial and OB is fast and robust up to a stimulation frequency of 10 Hz. These provide reference for the application of optogenetics in exploring the physiological and pathological mechanisms of olfaction [21].

Interesting is that optogenetic induction of different activation time patterns (0.04 Hz, 1 Hz, and continuous light) in ORNs results in different behavioral outputs (running speed and distance), indicating temporal resolution characteristics of olfaction [22]. Moreover, Clark et al. used optogenetics to simulate different ORN neurons and observed resultant changes in larval behavior, specifically the median run length. Subsequently, they classified seven ORNs into two categories: increment-ORNs, which include ORN::7a, ORN::42b, ORN::45a, ORN::47a, and ORN::67b; and decrement-ORNs, which comprise ORN::42a and ORN::45b [22]. Increment-ORNs drive behavior changes in response to light increments (if there is a significant drop in run length when light changed from OFF to ON, the ORN is considered to be active in repulsive responses), while decrement-ORNs drive behavior changes in response to light decrements (if there is a significant drop in run length when light changed from ON to OFF, the ORN is considered to be active in attractive responses). True ON neurons show responses to stimulus increases, while OFF neurons show responses to stimulus decreases. If ON and OFF-ORNs provide responses to increases and decreases in odor concentration, respectively, this bidirectional effect may help to assess odor flow in real time and distinguish between high and low concentrations of odors, thus fine-tuning tracking decisions. Currently, this feature has only been found in insects [23, 24]. Excitingly, it has been found that light stimulation activating different ORNs expressing ChR2 in the fruit fly olfactory system can induce either repellent or attractive behaviors, respectively. This suggests that different behavioral types have distinct sensory neuron bases, and these sensory neurons are useful targets for manipulating olfactory behavior [25, 26].

### 2.2. Application in OB

The sense of smell allows us to detect odors in the external environment [27, 28]. Odorants bind to olfactory receptors on ORNs in the nasal epithelium, which sends signals to the OB [29, 30]. The OB is the primary processing center for olfactory information [31, 32]. It has a unique laminar structure consisting of five layers from outer to inner: glomerular layer (GL), external plexiform layer (EPL), mitral cell (MC) layer (MCL), internal plexiform layer (IPL), and granule cell (GC) layer (GCL). OB neurons include projection neurons and interneurons. Projection neurons, which include MCs and tufted cells (TCs), are the major excitatory output neurons, while interneurons are predominantly gamma-aminobutyric acid (GABA) ergic and can be further classified by location as periglomerular cells (PGs) in the GL, GCs in the GCL, and short axon (SA) cells throughout the OB [30]. Olfactory glomeruli in the GL are the most basic functional units for processing olfactory information in the OB. Within the glomeruli, ORNs form synapses with mitral or TCs, which further process the information from the ORNs.

Oscillation of the OB is critical for odor perception in insects and mammals, and odor processing is associated with beta and gamma oscillations [33]. Related research has found that ErbB4-positive (ErbB4+) cells in the OB are a novel type of interneuron that enhances theta oscillation when activated by photogenetics in GL-ErbB4+ neurons and increases gamma oscillation when activated in GCL-ErbB4+ neurons [34, 35]. This confirms that the activity of ErbB4+ neurons and the neuregulin-1 (NRG1, the ligand of ErbB4)-ErbB4 signaling pathway are essential for oscillation and olfaction in the bulb, which has profound implications for revealing potential mechanisms of related olfactory disorders [35]. Odors evoke responses in glomeruli, creating combinatorial representations that encode odor identity [36, 37]. This representation not only differs between different odors but also varies with concentration for a given odor [38–40]. Low concentrations of an odor elicit activity only in the most sensitive glomeruli, while increasing concentration recruits additional, less sensitive glomeruli. One study proposes the first code for odor identity, defined by the subset of olfactory glomeruli that are earliest activated and most sensitive to each odor, and verifies it through an optogenetic masking paradigm [12]. The masking paradigm creates a time-controlled masking stimulus in an odor identification task. It was found that optogenetic masking stimuli given after odor identification did not impair odor recognition, but those given before odor identification did damage odor discrimination, confirming the first code for odor identity. Furthermore, it was reported that by optogenetically stimulating multiple glomeruli, sensory inputs were formed, and different firing patterns were detected in MCs and TCs. TC exhibited robust, widespread, and sustained spike-time synchrony at fast and slow gamma frequencies, while MC synchronization was weaker and concentrated at slow gamma frequencies [41]. This revealed a parallel processing mechanism for olfactory sensory information through synchronized firing of TCs and MCs in the OB. The specific reasons may be that a series of interneurons in the OB mediate differential inhibition between TCs and MCs, and further research is needed on how interneurons promote rapid network oscillation in the OB. In adult rodents, the OB is supplied with new inhibitory interneurons, which shape the information carried by MCs outside the OB. Forest et al. demonstrated that adult-born neurons play a crucial role in regulating an animal's discriminative ability through perceptual learning [42]. They discovered that training in sensory learning about odors leads to an increase in the number of adult neurons in the OB, which subsequently enhances the ability to distinguish that specific odor. Additionally, when adult-born neurons were inhibited optogenetically, it prevented the discrimination of odors following perceptual learning. This result suggests that adult-born neurons significantly contribute to behavioral adaptation in olfaction.

The OB also receives many centrifugal fibers from cortical structures, including the anterior olfactory nucleus (AON), olfactory cortex, piriform cortex (PCX), locus coeruleus (LC), and midbrain (interfascicular nucleus), which terminate in the GCL or GL of the OB. The impact of these centrifugal fibers on sensory information processing is mainly inhibitory, acting as feedback loops. The major source of cortical feedback projections to the OB is the AON, a ring-like cortical structure located caudal to the OB and rostral to the PCX, which provides direct excitatory inputs to inhibitory interneurons and MCs [43, 44]. Medinaceli Quintela et al. found that optogenetic activation of the AON could inhibit odor-evoked MC firing and odor responses, revealing the role of the AON in olfactory information transmission [45]. In addition, Aqrabawi et al. found that the pars medialis (mAON) neuropil transmitted primarily to the GCL of the OB, with lesser transmission to the GL [46]. The ventral hippocampus (vHPC) was identified as a potential cortical input source for mAON, and optogenetic activation of vHPC input to mAON decreased olfactory sensitivity, indicating that vHPC is a candidate region for high-order modulation of olfactory sensitivity through mAON [47]. However, the functional significance of other neocortix that alters olfaction through mAON remains to be further explored. Studies have shown that stimulation of LC fibers can result in the release of dopamine and norepinephrine [47]. Linster et al. injected lentivirus expressing eNpHR3.0 under the hSyn promoter into the LC of mice and placed optical fibers in the OB to optogenetically suppress norepinephrine. They found that acute suppression of norepinephrine could change the duration, stability, and specificity of olfactory memory, indicating that the presence of norepinephrine in the OB makes olfactory memory more stable [48].

### 2.3. Application in Olfactory Center

The axons of mitral/TCs form the olfactory tract, which relays information to various regions of the brain, including the PCX, amygdala, and olfactory tubercle (OT). Sensory cues can dramatically influence feeding behavior. Olfaction has been shown to impact both food intake and metabolism [49]. Interestingly, hypothalamic feeding-related regions such as the arcuate nucleus (ARC), lateral hypothalamus (LH), and paraventricular nucleus (PVN) are regulated by the perception of food odors [50–52]. However, the circuitry mechanisms by which olfactory information is transmitted to hypothalamic nodes involved in feeding are not yet fully understood. Optogenetics allows for precise spatial and temporal control of neural activity. To investigate the circuitry relaying sensory cues to the hypothalamus, Swanson et al. manipulated circuit activity in a cell-type-specific manner using optogenetic methods and found that the basal forebrain (BF) to lateral habenula (LHb) circuit responds to various sensory cues, including odor-induced food aversion [53]. Activation of this neural circuit drives strong aversion and can suppress feeding drive in fasting states. Studying the operation and anatomical connections of this circuit may help us better understand and treat diseases such as obesity and eating disorders.

For rodents, reward-related odor perception is critical for food foraging and reproduction [54]. The formation of odor preference should involve the interaction between the olfactory and reward systems. OT belongs to the olfactory system and receives direct and intensive inputs from the main OB and other olfactory cortices such as AON and PCX [55]. At the same time, as part of the ventral striatal reward circuit, OT is also heavily dominated by dopaminergic neurons from the ventral tegmental area (VTA) [56]. As it belongs to both the olfactory and reward circuits, the medial OT (mOT) plays a critical role in the formation of odor preferences [57]. Zhang et al. found that optogenetic activation of the VTA (DAergic)-mOT pathway can generate spatial/location preferences [58]. Combining this activation with odor stimulation not only generates preference for neutral odors but also eliminates avoidance for aversive odors [58]. In addition, the inactivation of VTA-DAergic neurons projecting to mOT or blocking dopamine receptors in the mOT can abolish previously formed odor preferences [58]. These results suggest that activation of the VTA (DAergic)-mOT pathway produces odor preferences and other types of preferences, making it a crucial factor in regulating the perception of given stimuli.

Impairment of smell can be caused by an injury to any part of the olfactory tract. Currently, most olfactory deficits, which are neuronally mediated, cannot be corrected. However, the concept of directly stimulating specific areas of the brain to activate or inhibit a sensory perception is not unprecedented. Recently, the use of optogenetic stimulation of the cochlea has been proposed as an alternative approach for hearing restoration [59]. These same principles that have contributed to the success of optogenetic hearing restoration could potentially be applied to treat anosmia, or loss of smell.

## 3. Application in Sensory Nervous System

The nose is innervated by the trigeminal nerve. The symptoms that rhinitis patients, such as AR and idiopathic rhinitis, typically experience are often the result of alterations in the nervous system. Due to the limited understanding of neural circuits and brain functional regions, we currently lack effective treatments that directly and specifically target these neural circuits. However, optogenetics has demonstrated immense potential in studying the structure, physiology, and mechanisms of the brain and may provide a potential treatment avenue for nasal diseases.

### 3.1. Application in Trigeminal Nerve Fiber

An intriguing phenomenon is observed in some individuals, where the effects produced by the same level of neural stimulation are more significant than those seen in healthy individuals. This heightened reactivity is known as nasal hyperresponsiveness [60]. Typically, afferent C-fibers are known to express receptors for various neural stimulations. These receptors can often be accurately classified as nociceptors. The ion channels known as the transient receptor potential (TRP) family are frequently expressed on afferent C-fibers and contribute to nociception. Specifically, Transient Receptor Potential Vanilloid 1 (TRPV1) and Transient Receptor Potential Ankyrin Repeat 1 (TRPA1) play critical roles in nasal hyperresponsiveness associated with rhinitis [61]. Activated directly by thermal, chemical, and mechanical stimuli, they result in sensations such as itching and sneezing [62]. It is recognized that TRPV1 density increases on sensory nerve fibers in patients with nasal hyperreactivity [63]. Several studies have investigated TRPV1 with the tool of optogenetics. For instance, Beaudry et al. used optogenetics to specifically deliver the excitatory opsin ChR2 to TRPV1 primary sensory neurons, selectively activating the major subset of C-fibers in adult mice in vivo [64]. Their results showed that transdermal blue light stimulation of transgenic mice expressing ChR2 in TRPV1 neurons generated behaviors such as paw withdrawal and paw licking, which assessed the function of TRPV1 in somatosensory transmission. Li et al. aimed to determine if there exists a noninvasive method with high specificity to inhibit nociception [65]. In their study, they used a TRPV1 channel promoter to selectively express inhibitory light-sensitive pump ArchT in nociceptive neurons by injecting a recombinant virus (AAV5-TRPV1-ArchT-eGFP). Their findings revealed that green light led to nociception inhibition in mice. Tochitsky et al. pointed out that for optogenetics to be more clinically applicable, the development of an endogenous protein-targeting photoswitch that confers light sensitivity on endogenous neuronal ion channels might be beneficial [66]. As a result, chemical optogenetic tools that blend chemistry and optogenetics are quickly evolving. Photochromic soluble ligands (PCLs) of various wild-type ion channels have been synthesized using chemical design methods to achieve light-controlled channel activity [67]. These PCLs can block and unlock the corresponding ion channel. The most typical example is the PCL of the TRPV1 channel. Frank et al. developed a toolkit of photoswitchable fatty acid analogues (FAAzos) that incorporate an azobenzene photoswitch along the FA chain [67]. By modifying the FAAzos to resemble capsaicin, they prepared a series of photolipids targeting TRPV1. Consequently, several azo-capsaicin derivatives emerged as photoswitchable agonists of TRPV1, which remain relatively inactive in the dark but become active under ultraviolet-A light. Blue light stimulation can swiftly reverse these effects. During the process, trigeminal neurons and C-fiber nociceptors can be precisely controlled by light.

TRPA1, a nociceptive cation (primarily Ca2+), plays a role in sensing cold temperatures below 17°C [68]. Consequently, cold stimuli stimulate sensory neurons and cause neurogenic inflammation. This phenomenon might explain why individuals with rhinitis are more susceptible to sneezing when inhaling cold air. One study demonstrated that an antagonist of TRPA1 (HC-030031) could reduce airway inflammation and hyperresponsiveness in a murine model of AR [69]. Using chemo-optogenetics, Lam et al. reported photoswitchable ligands for the TRPA1 channel, such as TRPswitch, and established their capability to optically control both the activation and deactivation of the TRPA1 channel using violet light and green light, respectively [70]. Notably, the TRPA1/TRPswitch chemo-optogenetic system has been proven capable of regulating the heartbeat in zebrafish [70]. Although the applicability of the TRPA1/TRPswitch chemo-optogenetic system to other in vivo and clinical applications remains unknown, it shows promise in regulating TRPA1 receptors located in nasal sensory nerve endings. On another note, Qiao et al. designed and synthesized a series of azobenzene and its analogues that can be controlled by light [71]. Under ultraviolet light, azobenzene derivatives activate TRPA1 channels in a concentration-dependent manner, leading to an inflow of calcium ions. The study found that after topical application of azobenzene compounds to the cheeks of mice and subsequent exposure to 365 nm light, the number of face wipes induced by capsaicin significantly decreased. The observed effect of azobenzene compounds under ultraviolet light is due to the desensitization of the TRPA1 channel. In the future, this system may potentially be used to modulate both TRPV1 and TRPA1, thereby helping reduce nasal hyperresponsiveness.

### 3.2. Application in Brain Regions

As previously discussed, nasal hyperresponsiveness in patients with rhinitis is characterized by heightened and consistent nociceptor activity. This increase indirectly regulates central nervous system (CNS) neurons, leading to greater excitability. This condition also results in an increased release of neurotransmitters/neuropeptides at the central terminal of afferent nerves, consequently heightening the synaptic efficacy of CNS neurons, a process often referred to as “central sensitization.” Central sensitization is frequently mentioned in the context of allergies, including AR. Studies suggest that central sensitization might contribute to the continuous urge to sneeze even when no physical irritant is present in the nasal passage [60]. As CNS neurons become sensitized, the signal of afferent fiber can be amplified or even qualitatively transformed [60]. Previous research has indicated that central sensitization entails enhanced responses to excitatory amino acids and diminished responses to inhibitory amino acids [72]. Mechanisms for these changes include the phosphorylation of N-methyl-D-aspartate receptors (NMDARs) [73] and *γ*-aminobutyric acid receptors (GABARs) [74], respectively. Davenport et al. developed a knockin mouse model enabling optical control of endogenous *α*5-subunit-containing *γ*-aminobutyric acid receptors (*α*5-GABARs) [75]. Results showed that blocking *α*5-GABARs could expedite NMDA receptor activation required for inducing more excitatory long-term potentiation (LTP), reflecting activity-dependent synaptic plasticity and central sensitization [76]. Laprell et al. synthesized a photoswitchable agonist, which is specific for NMDARs [77]. It can be rapidly activated by irradiation with a 360–375 nm light band and swiftly deactivated with a 405–460 nm light band. Additionally, Berlin et al. engineered a set of photoswitchable GluN subunits comprising NMDA receptors. They found that photo-antagonism of GluN2A alone, or combined with photo-antagonism of GluN1a, reversibly blocks excitatory synaptic currents and prevents the induction of LTP [78]. In the future, chemo-optogenetic may be possible to modulate NMDAR as well as GABAR to reduce central sensitization and improve rhinitis symptoms.

## 4. Current Limitations and Future Perspective

Despite its rapid advancement, optogenetics still faces several challenges and limitations before its potential can be fully harnessed and translated clinically for controlling rhinitis. Firstly, the continuous delivery of light to target cells or tissue regions may lead to heating effects, tissue damage, and off-target cellular activities [79]. Prolonged exposure to light can reduce cell viability and induce transcriptional activation and oxidative stress responses in various cell types, including immune cells [80]. Besides, high expression levels and extended duration of opsin expression might result in dark activity (autoactivation) and unwanted cellular toxicity, both of which can alter cell physiology [79]. Furthermore, most current light delivery systems are invasive, tethering animals with optical fibers, which restricts animal behavior and compromises the reproducibility of behavioral test results. Invasive implantation of microdevices can also stimulate prolonged inflammation, which could affect cellular activity and introduce confounding factors into changes in tissue physiology. To enhance the clinical applicability of optogenetics, chemo-optogenetic tools have been rapidly developed as a complementary approach. The utilization of vertebrate protein actuators might mitigate the risk of immunological reactions due to prolonged expression of exogenous proteins.

At present, optogenetics in nose control is investigated mostly in rodent models. Tackling olfaction loss or treating eye diseases such as retinitis pigmentosa may represent easier paths towards the clinical applicability of optogenetics, as minimally invasive interventions are readily possible [81]. Currently, four companies have progressed optogenetic retinal gene therapies into clinical trials [82]. Sahel et al. were the first to report a case of partial recovery of visual function following optogenetic therapy [83]. A blind patient who received an intraocular injection of an adeno-associated viral vector encoding ChrimsonR was able to visually detect objects with light stimulation via specially designed goggles. Such advancements underscore the promising future of optogenetics for clinical applications, including its potential use in nasal neuromodulation.

## 5. Conclusion

This review summarizes recent advancements and potential applications of optogenetics in nasal neuromodulation. Optogenetics serves as a powerful tool for investigating the neural circuitry of olfaction and offers a promising approach for manipulating neurons, including the olfactory nerve and the sensory nervous system, to achieve coordinated nasal modulation. However, certain limitations of optogenetics, such as the side effects of continuous light exposure and the safety concerns related to light delivery devices, hinder the translation of optogenetic neuromodulation into clinical practice. Despite these challenges, several pilot clinical studies utilizing optogenetics are currently underway, indicating its potential for clinical application. In the future, optogenetic neuromodulation may also be utilized for nasal control in clinical settings.

## Data Availability

The authors have nothing to report.
